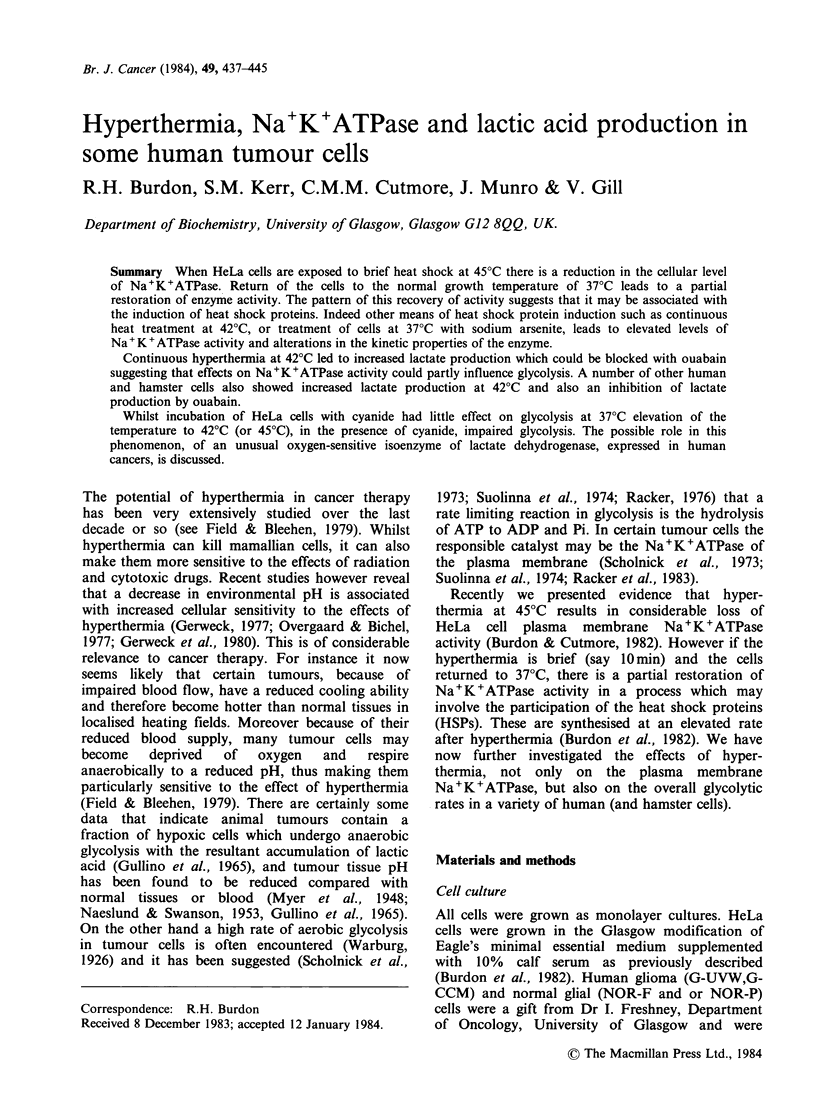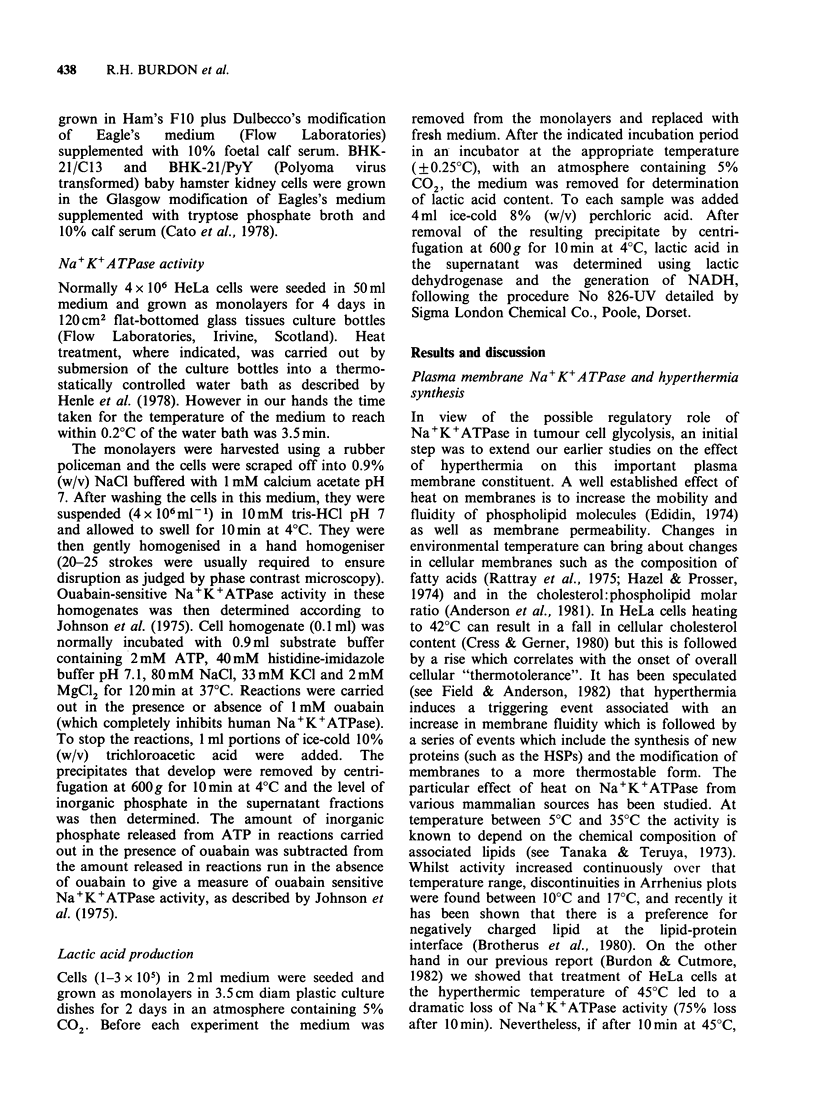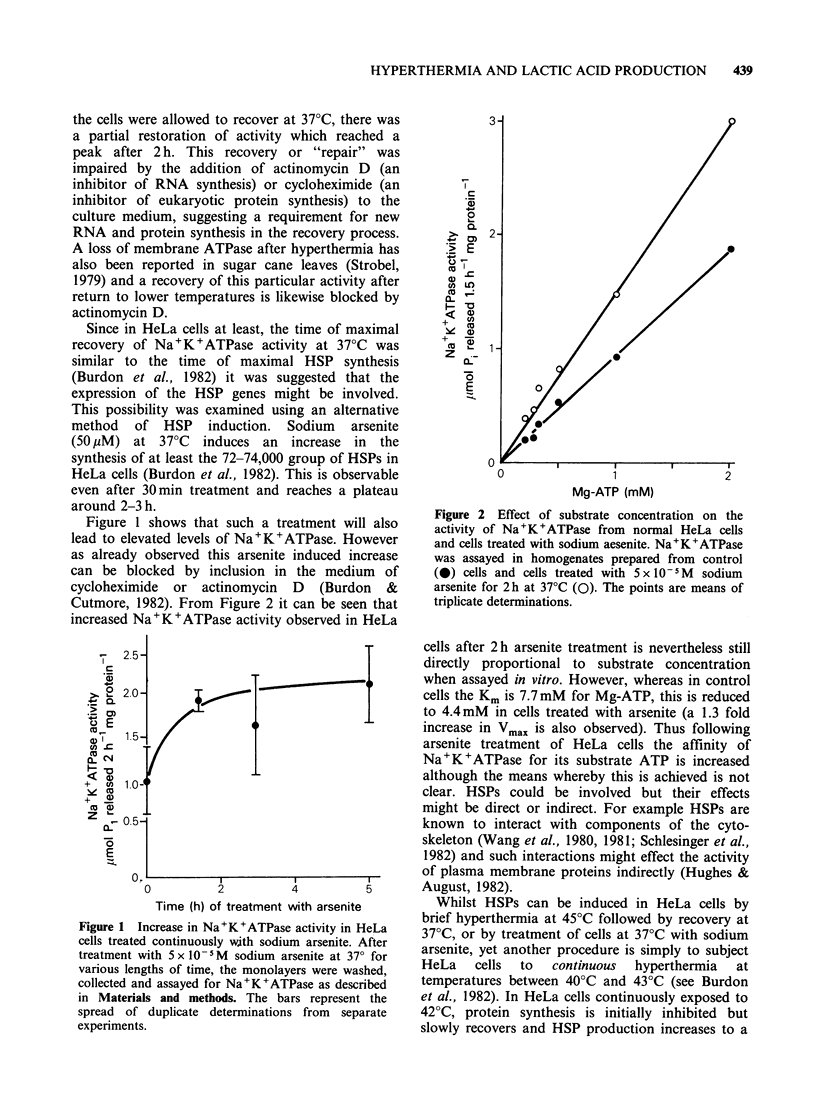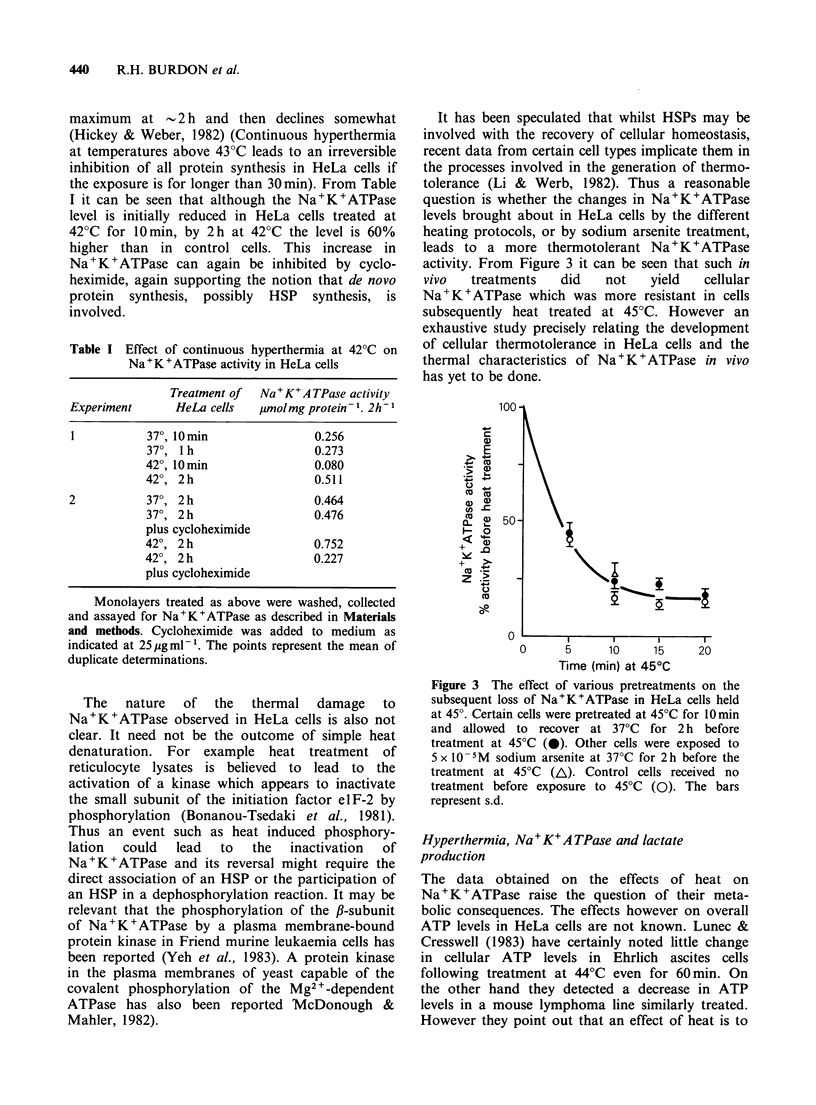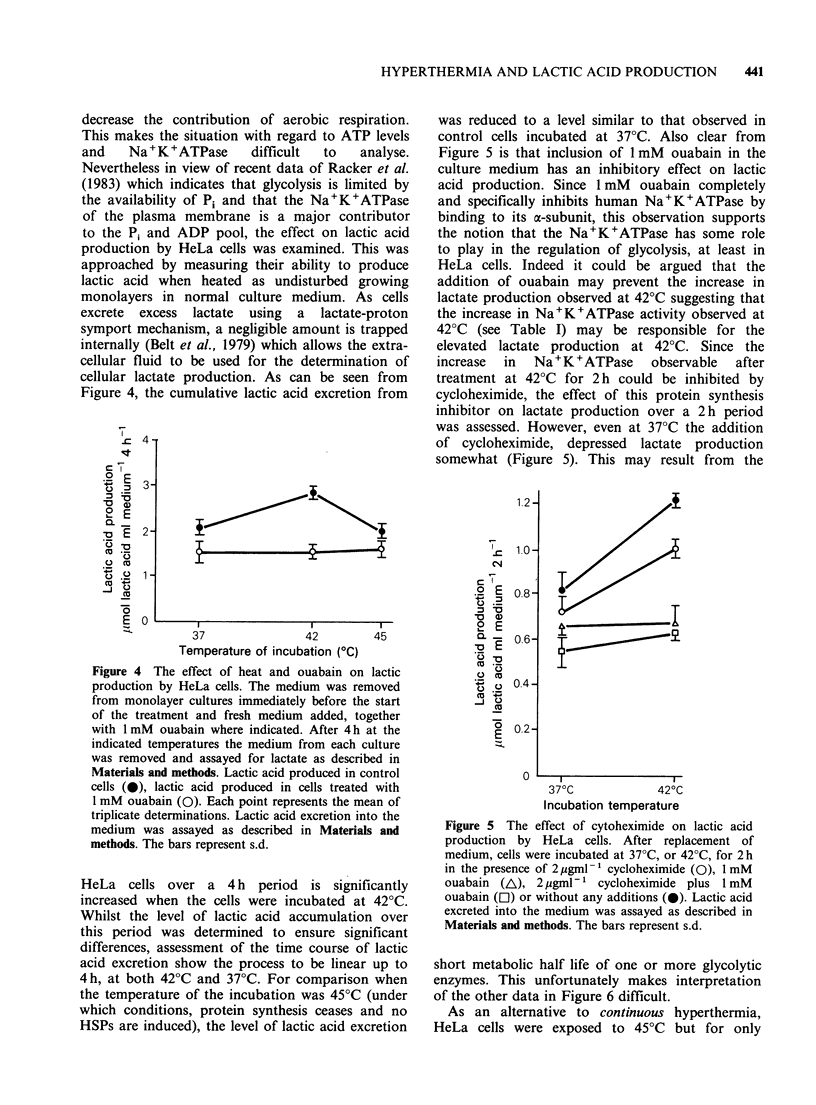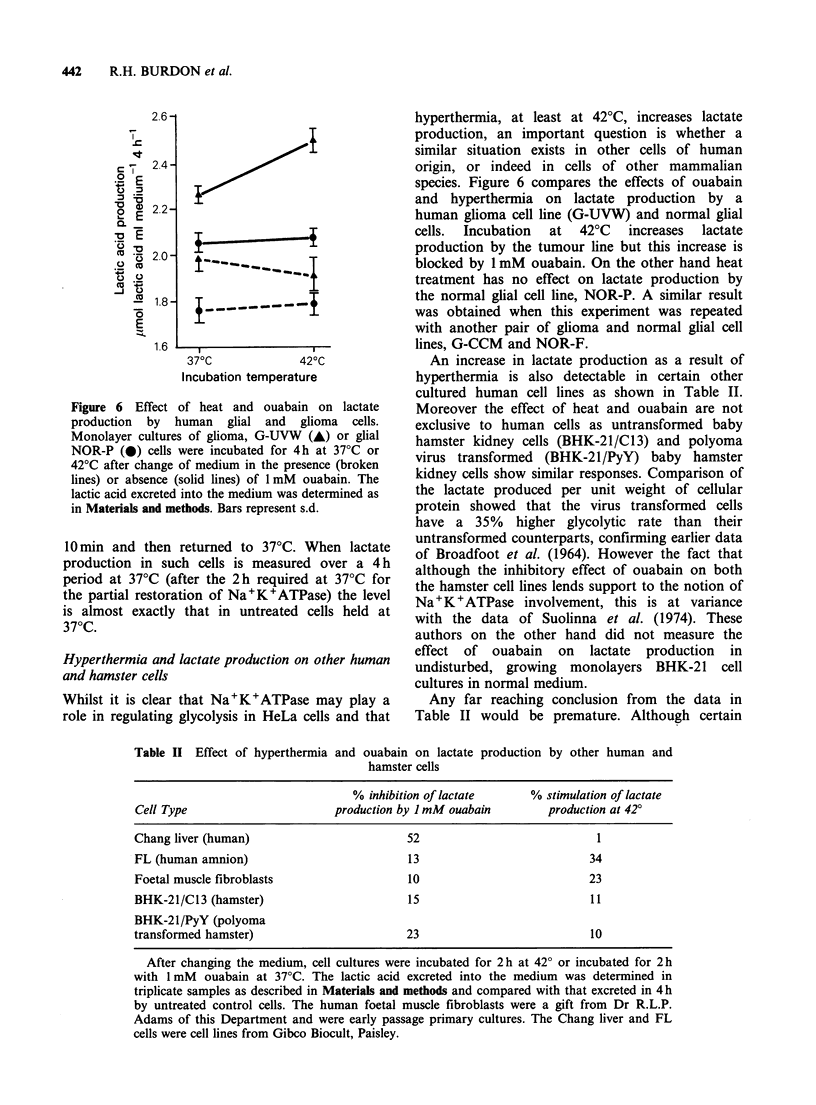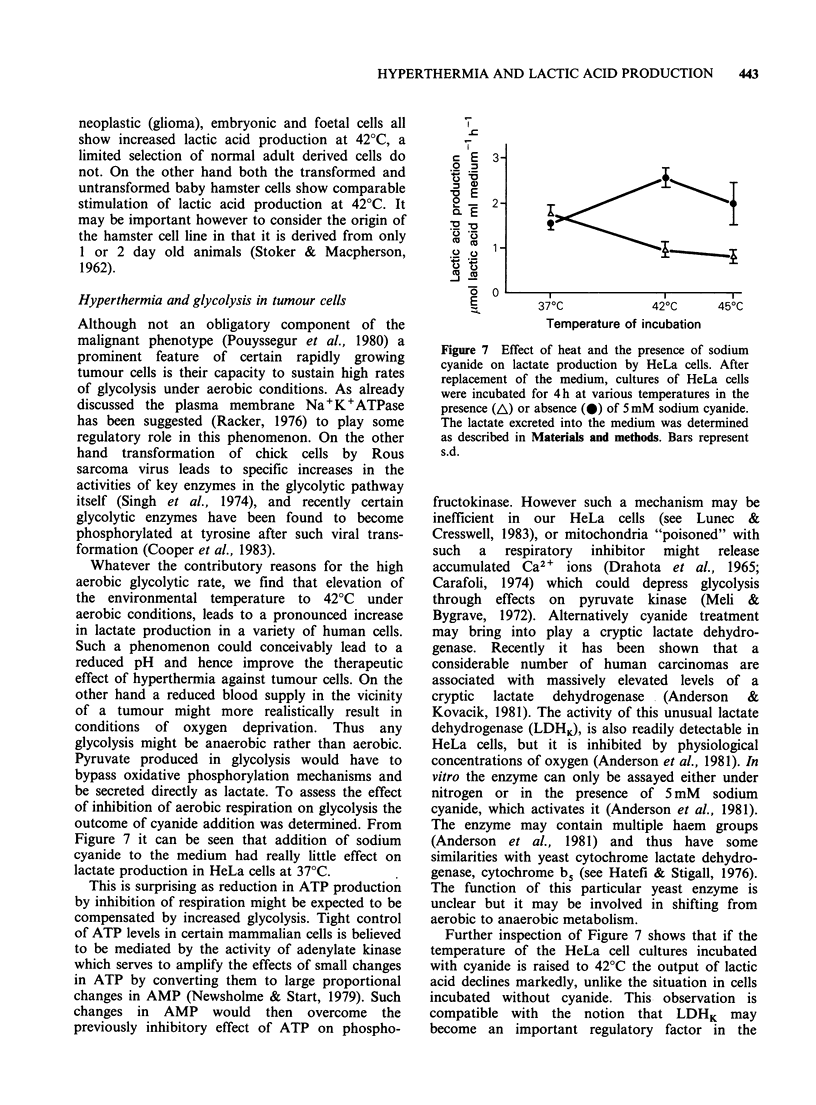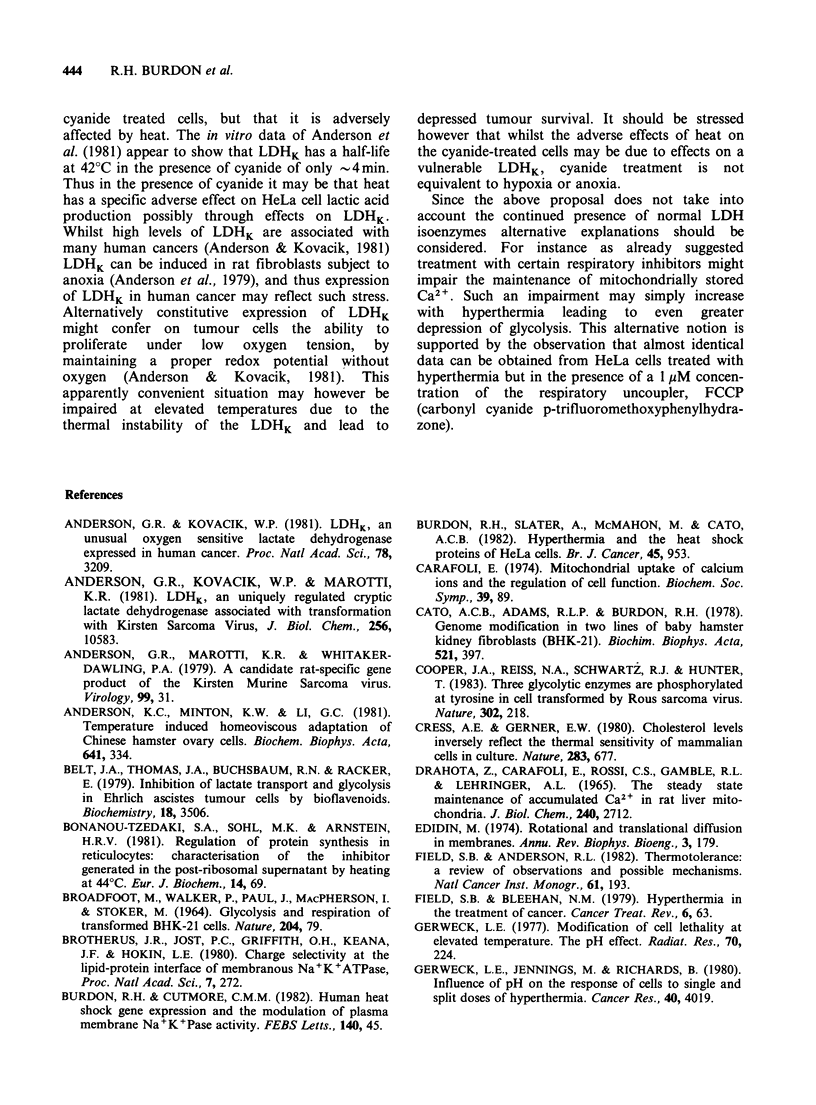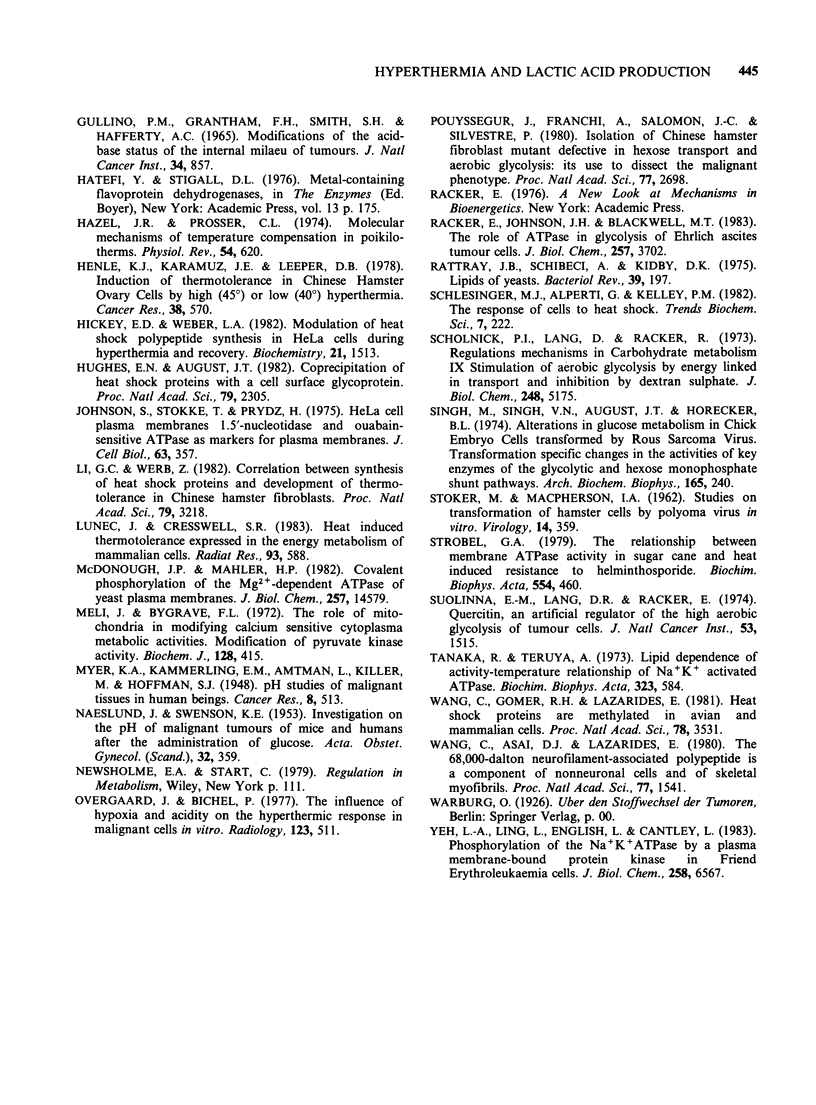# Hyperthermia, Na+K+ATPase and lactic acid production in some human tumour cells.

**DOI:** 10.1038/bjc.1984.70

**Published:** 1984-04

**Authors:** R. H. Burdon, S. M. Kerr, C. M. Cutmore, J. Munro, V. Gill

## Abstract

When HeLa cells are exposed to brief heat shock at 45 degrees C there is a reduction in the cellular level of Na+K+ATPase. Return of the cells to the normal growth temperature of 37 degrees C leads to a partial restoration of enzyme activity. The pattern of this recovery of activity suggests that it may be associated with the induction of heat shock proteins. Indeed other means of heat shock protein induction such as continuous heat treatment at 42 degrees C, or treatment of cells at 37 degrees C with sodium arsenite, leads to elevated levels of Na+K+ATPase activity and alterations in the kinetic properties of the enzyme. Continuous hyperthermia at 42 degrees C led to increased lactate production which could be blocked with ouabain suggesting that effects on Na+K+ATPase activity could partly influence glycolysis. A number of other human and hamster cells also showed increased lactate production at 42 degrees C and also an inhibition of lactate production by ouabain. Whilst incubation of HeLa cells with cyanide had little effect on glycolysis at 37 degrees C elevation of the temperature to 42 degrees C (or 45 degrees C), in the presence of cyanide, impaired glycolysis. The possible role in this phenomenon, of an unusual oxygen-sensitive isoenzyme of lactate dehydrogenase, expressed in human cancers, is discussed.


*Br. J. Cancer* (1984), **49**, 437–445

### Hyperthermia, $Na^+K^+$ATPase and lactic acid production in some human tumour cells

R.H. Burdon, S.M. Kerr, C.M.M. Cutmore, J. Munro & V. Gill

*Department of Biochemistry, University of Glasgow, Glasgow G12 8QQ, UK.*

**Summary** When HeLa cells are exposed to brief heat shock at 45°C there is a reduction in the cellular level of $Na^+K^+$ATPase. Return of the cells to the normal growth temperature of 37°C leads to a partial restoration of enzyme activity. The pattern of this recovery of activity suggests that it may be associated with the induction of heat shock proteins. Indeed other means of heat shock protein induction such as continuous heat treatment at 42°C, or treatment of cells at 37°C with sodium arsenite, leads to elevated levels of $Na^+K^+$ATPase activity and alterations in the kinetic properties of the enzyme.

Continuous hyperthermia at 42°C led to increased lactate production which could be blocked with ouabain suggesting that effects on $Na^+K^+$ATPase activity could partly influence glycolysis. A number of other human and hamster cells also showed increased lactate production at 42°C and also an inhibition of lactate production by ouabain.

Whilst incubation of HeLa cells with cyanide had little effect on glycolysis at 37°C elevation of the temperature to 42°C (or 45°C), in the presence of cyanide, impaired glycolysis. The possible role in this phenomenon, of an unusual oxygen-sensitive isoenzyme of lactate dehydrogenase, expressed in human cancers, is discussed.

The potential of hyperthermia in cancer therapy has been very extensively studied over the last decade or so (see Field & Bleehen, 1979). Whilst hyperthermia can kill mamallian cells, it can also make them more sensitive to the effects of radiation and cytotoxic drugs. Recent studies however reveal that a decrease in environmental pH is associated with increased cellular sensitivity to the effects of hyperthermia (Gerweck, 1977; Overgaard & Bichel, 1977; Gerweck *et al.*, 1980). This is of considerable relevance to cancer therapy. For instance it now seems likely that certain tumours, because of impaired blood flow, have a reduced cooling ability and therefore become hotter than normal tissues in localised heating fields. Moreover because of their reduced blood supply, many tumour cells may become deprived of oxygen and respire anaerobically to a reduced pH, thus making them particularly sensitive to the effect of hyperthermia (Field & Bleehen, 1979). There are certainly some data that indicate animal tumours contain a fraction of hypoxic cells which undergo anaerobic glycolysis with the resultant accumulation of lactic acid (Gullino *et al.*, 1965), and tumour tissue pH has been found to be reduced compared with normal tissues or blood (Myer *et al.*, 1948; Naeslund & Swanson, 1953, Gullino *et al.*, 1965). On the other hand a high rate of aerobic glycolysis in tumour cells is often encountered (Warburg, 1926) and it has been suggested (Scholnick *et al.*, 1973; Suolinna *et al.*, 1974; Racker, 1976) that a rate limiting reaction in glycolysis is the hydrolysis of ATP to ADP and Pi. In certain tumour cells the responsible catalyst may be the $Na^+K^+$ATPase of the plasma membrane (Scholnick *et al.*, 1973; Suolinna *et al.*, 1974; Racker *et al.*, 1983).

Recently we presented evidence that hyperthermia at 45°C results in considerable loss of HeLa cell plasma membrane $Na^+K^+$ATPase activity (Burdon & Cutmore, 1982). However if the hyperthermia is brief (say 10 min) and the cells returned to 37°C, there is a partial restoration of $Na^+K^+$ATPase activity in a process which may involve the participation of the heat shock proteins (HSPs). These are synthesised at an elevated rate after hyperthermia (Burdon *et al.*, 1982). We have now further investigated the effects of hyperthermia, not only on the plasma membrane $Na^+K^+$ATPase, but also on the overall glycolytic rates in a variety of human (and hamster cells).

### Materials and methods

### *Cell culture*

All cells were grown as monolayer cultures. HeLa cells were grown in the Glasgow modification of Eagle's minimal essential medium supplemented with 10% calf serum as previously described (Burdon *et al.*, 1982). Human glioma (G-UVW,G-CCM) and normal glial (NOR-F and or NOR-P) cells were a gift from Dr I. Freshney, Department of Oncology, University of Glasgow and were

Correspondence: R.H. Burdon
Received 8 December 1983; accepted 12 January 1984.

© The Macmillan Press Ltd., 1984

grown in Ham's F10 plus Dulbecco's modification of Eagle's medium (Flow Laboratories) supplemented with 10% foetal calf serum. BHK-21/C13 and BHK-21/PyY (Polyoma virus transformed) baby hamster kidney cells were grown in the Glasgow modification of Eagles's medium supplemented with tryptose phosphate broth and 10% calf serum (Cato *et al.*, 1978).

*$Na^+K^+$ATPase activity*

Normally $4 \times 10^6$ HeLa cells were seeded in 50 ml medium and grown as monolayers for 4 days in 120 cm$^2$ flat-bottomed glass tissues culture bottles (Flow Laboratories, Irivine, Scotland). Heat treatment, where indicated, was carried out by submersion of the culture bottles into a thermostatically controlled water bath as described by Henle *et al.* (1978). However in our hands the time taken for the temperature of the medium to reach within 0.2°C of the water bath was 3.5 min.

The monolayers were harvested using a rubber policeman and the cells were scraped off into 0.9% (w/v) NaCl buffered with 1 mM calcium acetate pH 7. After washing the cells in this medium, they were suspended ($4 \times 10^6$ ml$^{-1}$) in 10 mM tris-HCl pH 7 and allowed to swell for 10 min at 4°C. They were then gently homogenised in a hand homogeniser (20–25 strokes were usually required to ensure disruption as judged by phase contrast microscopy). Ouabain-sensitive $Na^+K^+$ATPase activity in these homogenates was then determined according to Johnson *et al.* (1975). Cell homogenate (0.1 ml) was normally incubated with 0.9 ml substrate buffer containing 2 mM ATP, 40 mM histidine-imidazole buffer pH 7.1, 80 mM NaCl, 33 mM KCl and 2 mM $MgCl_2$ for 120 min at 37°C. Reactions were carried out in the presence or absence of 1 mM ouabain (which completely inhibits human $Na^+K^+$ATPase). To stop the reactions, 1 ml portions of ice-cold 10% (w/v) trichloroacetic acid were added. The precipitates that develop were removed by centrifugation at 600 *g* for 10 min at 4°C and the level of inorganic phosphate in the supernatant fractions was then determined. The amount of inorganic phosphate released from ATP in reactions carried out in the presence of ouabain was subtracted from the amount released in reactions run in the absence of ouabain to give a measure of ouabain sensitive $Na^+K^+$ATPase activity, as described by Johnson *et al.* (1975).

*Lactic acid production*

Cells ($1–3 \times 10^5$) in 2 ml medium were seeded and grown as monolayers in 3.5 cm diam plastic culture dishes for 2 days in an atmosphere containing 5% $CO_2$. Before each experiment the medium was removed from the monolayers and replaced with fresh medium. After the indicated incubation period in an incubator at the appropriate temperature (±0.25°C), with an atmosphere containing 5% $CO_2$, the medium was removed for determination of lactic acid content. To each sample was added 4 ml ice-cold 8% (w/v) perchloric acid. After removal of the resulting precipitate by centrifugation at 600 *g* for 10 min at 4°C, lactic acid in the supernatant was determined using lactic dehydrogenase and the generation of NADH, following the procedure No 826-UV detailed by Sigma London Chemical Co., Poole, Dorset.

### Results and discussion

*Plasma membrane $Na^+K^+$ATPase and hyperthermia synthesis*

In view of the possible regulatory role of $Na^+K^+$ATPase in tumour cell glycolysis, an initial step was to extend our earlier studies on the effect of hyperthermia on this important plasma membrane constituent. A well established effect of heat on membranes is to increase the mobility and fluidity of phospholipid molecules (Edidin, 1974) as well as membrane permeability. Changes in environmental temperature can bring about changes in cellular membranes such as the composition of fatty acids (Rattray *et al.*, 1975; Hazel & Prosser, 1974) and in the cholesterol:phospholipid molar ratio (Anderson *et al.*, 1981). In HeLa cells heating to 42°C can result in a fall in cellular cholesterol content (Cress & Gerner, 1980) but this is followed by a rise which correlates with the onset of overall cellular "thermotolerance". It has been speculated (see Field & Anderson, 1982) that hyperthermia induces a triggering event associated with an increase in membrane fluidity which is followed by a series of events which include the synthesis of new proteins (such as the HSPs) and the modification of membranes to a more thermostable form. The particular effect of heat on $Na^+K^+$ATPase from various mammalian sources has been studied. At temperature between 5°C and 35°C the activity is known to depend on the chemical composition of associated lipids (see Tanaka & Teruya, 1973). Whilst activity increased continuously over that temperature range, discontinuities in Arrhenius plots were found between 10°C and 17°C, and recently it has been shown that there is a preference for negatively charged lipid at the lipid-protein interface (Brotherus *et al.*, 1980). On the other hand in our previous report (Burdon & Cutmore, 1982) we showed that treatment of HeLa cells at the hyperthermic temperature of 45°C led to a dramatic loss of $Na^+K^+$ATPase activity (75% loss after 10 min). Nevertheless, if after 10 min at 45°C, the cells were allowed to recover at 37°C, there was a partial restoration of activity which reached a peak after 2 h. This recovery or "repair" was impaired by the addition of actinomycin D (an inhibitor of RNA synthesis) or cycloheximide (an inhibitor of eukaryotic protein synthesis) to the culture medium, suggesting a requirement for new RNA and protein synthesis in the recovery process. A loss of membrane ATPase after hyperthermia has also been reported in sugar cane leaves (Strobel, 1979) and a recovery of this particular activity after return to lower temperatures is likewise blocked by actinomycin D.

Since in HeLa cells at least, the time of maximal recovery of $Na^+K^+$ATPase activity at 37°C was similar to the time of maximal HSP synthesis (Burdon *et al.*, 1982) it was suggested that the expression of the HSP genes might be involved. This possibility was examined using an alternative method of HSP induction. Sodium arsenite (50 μM) at 37°C induces an increase in the synthesis of at least the 72–74,000 group of HSPs in HeLa cells (Burdon *et al.*, 1982). This is observable even after 30 min treatment and reaches a plateau around 2–3 h.

Figure 1 shows that such a treatment will also lead to elevated levels of $Na^+K^+$ATPase. However as already observed this arsenite induced increase can be blocked by inclusion in the medium of cycloheximide or actinomycin D (Burdon & Cutmore, 1982). From Figure 2 it can be seen that increased $Na^+K^+$ATPase activity observed in HeLa cells after 2 h arsenite treatment is nevertheless still directly proportional to substrate concentration when assayed *in vitro*. However, whereas in control cells the $K_m$ is 7.7 mM for Mg-ATP, this is reduced to 4.4 mM in cells treated with arsenite (a 1.3 fold increase in $V_{max}$ is also observed). Thus following arsenite treatment of HeLa cells the affinity of $Na^+K^+$ATPase for its substrate ATP is increased although the means whereby this is achieved is not clear. HSPs could be involved but their effects might be direct or indirect. For example HSPs are known to interact with components of the cytoskeleton (Wang *et al.*, 1980, 1981; Schlesinger *et al.*, 1982) and such interactions might effect the activity of plasma membrane proteins indirectly (Hughes & August, 1982).

Whilst HSPs can be induced in HeLa cells by brief hyperthermia at 45°C followed by recovery at 37°C, or by treatment of cells at 37°C with sodium arsenite, yet another procedure is simply to subject HeLa cells to *continuous* hyperthermia at temperatures between 40°C and 43°C (see Burdon *et al.*, 1982). In HeLa cells continuously exposed to 42°C, protein synthesis is initially inhibited but slowly recovers and HSP production increases to a

**Figure 1** Increase in $Na^+K^+$ATPase activity in HeLa cells treated continuously with sodium arsenite. After treatment with $5 \times 10^{-5}$ M sodium arsenite at 37° for various lengths of time, the monolayers were washed, collected and assayed for $Na^+K^+$ATPase as described in **Materials and methods.** The bars represent the spread of duplicate determinations from separate experiments.

**Figure 2** Effect of substrate concentration on the activity of $Na^+K^+$ATPase from normal HeLa cells and cells treated with sodium aesenite. $Na^+K^+$ATPase was assayed in homogenates prepared from control (●) cells and cells treated with $5 \times 10^{-5}$ M sodium arsenite for 2 h at 37°C (○). The points are means of triplicate determinations.

maximum at ~2 h and then declines somewhat (Hickey & Weber, 1982) (Continuous hyperthermia at temperatures above 43°C leads to an irreversible inhibition of all protein synthesis in HeLa cells if the exposure is for longer than 30 min). From Table I it can be seen that although the $Na^+K^+$ATPase level is initially reduced in HeLa cells treated at 42°C for 10 min, by 2 h at 42°C the level is 60% higher than in control cells. This increase in $Na^+K^+$ATPase can again be inhibited by cycloheximide, again supporting the notion that *de novo* protein synthesis, possibly HSP synthesis, is involved.

**Table I** Effect of continuous hyperthermia at 42°C on $Na^+K^+$ATPase activity in HeLa cells

| *Experiment* | *Treatment of HeLa cells* | *$Na^+K^+$ATPase activity $\mu mol\,mg\,protein^{-1}.\,2h^{-1}$* |
|---|---|---|
| 1 | 37°, 10 min | 0.256 |
| | 37°, 1 h | 0.273 |
| | 42°, 10 min | 0.080 |
| | 42°, 2 h | 0.511 |
| 2 | 37°, 2 h | 0.464 |
| | 37°, 2 h plus cycloheximide | 0.476 |
| | 42°, 2 h | 0.752 |
| | 42°, 2 h plus cycloheximide | 0.227 |

Monolayers treated as above were washed, collected and assayed for $Na^+K^+$ATPase as described in **Materials and methods**. Cycloheximide was added to medium as indicated at $25\,\mu g\,ml^{-1}$. The points represent the mean of duplicate determinations.

The nature of the thermal damage to $Na^+K^+$ATPase observed in HeLa cells is also not clear. It need not be the outcome of simple heat denaturation. For example heat treatment of reticulocyte lysates is believed to lead to the activation of a kinase which appears to inactivate the small subunit of the initiation factor elF-2 by phosphorylation (Bonanou-Tsedaki *et al.*, 1981). Thus an event such as heat induced phosphorylation could lead to the inactivation of $Na^+K^+$ATPase and its reversal might require the direct association of an HSP or the participation of an HSP in a dephosphorylation reaction. It may be relevant that the phosphorylation of the $\beta$-subunit of $Na^+K^+$ATPase by a plasma membrane-bound protein kinase in Friend murine leukaemia cells has been reported (Yeh *et al.*, 1983). A protein kinase in the plasma membranes of yeast capable of the covalent phosphorylation of the $Mg^{2+}$-dependent ATPase has also been reported McDonough & Mahler, 1982).

It has been speculated that whilst HSPs may be involved with the recovery of cellular homeostasis, recent data from certain cell types implicate them in the processes involved in the generation of thermotolerance (Li & Werb, 1982). Thus a reasonable question is whether the changes in $Na^+K^+$ATPase levels brought about in HeLa cells by the different heating protocols, or by sodium arsenite treatment, leads to a more thermotolerant $Na^+K^+$ATPase activity. From Figure 3 it can be seen that such *in vivo* treatments did not yield cellular $Na^+K^+$ATPase which was more resistant in cells subsequently heat treated at 45°C. However an exhaustive study precisely relating the development of cellular thermotolerance in HeLa cells and the thermal characteristics of $Na^+K^+$ATPase *in vivo* has yet to be done.

**Figure 3** The effect of various pretreatments on the subsequent loss of $Na^+K^+$ATPase in HeLa cells held at 45°. Certain cells were pretreated at 45°C for 10 min and allowed to recover at 37°C for 2 h before treatment at 45°C (●). Other cells were exposed to $5\times10^{-5}$M sodium arsenite at 37°C for 2 h before the treatment at 45°C (△). Control cells received no treatment before exposure to 45°C (○). The bars represent s.d.

### *Hyperthermia, $Na^+K^+$ATPase and lactate production*

The data obtained on the effects of heat on $Na^+K^+$ATPase raise the question of their metabolic consequences. The effects however on overall ATP levels in HeLa cells are not known. Lunec & Cresswell (1983) have certainly noted little change in cellular ATP levels in Ehrlich ascites cells following treatment at 44°C even for 60 min. On the other hand they detected a decrease in ATP levels in a mouse lymphoma line similarly treated. However they point out that an effect of heat is to decrease the contribution of aerobic respiration. This makes the situation with regard to ATP levels and $Na^+K^+$ATPase difficult to analyse. Nevertheless in view of recent data of Racker *et al.* (1983) which indicates that glycolysis is limited by the availability of $P_i$ and that the $Na^+K^+$ATPase of the plasma membrane is a major contributor to the $P_i$ and ADP pool, the effect on lactic acid production by HeLa cells was examined. This was approached by measuring their ability to produce lactic acid when heated as undisturbed growing monolayers in normal culture medium. As cells excrete excess lactate using a lactate-proton symport mechanism, a negligible amount is trapped internally (Belt *et al.*, 1979) which allows the extracellular fluid to be used for the determination of cellular lactate production. As can be seen from Figure 4, the cumulative lactic acid excretion from

**Figure 4** The effect of heat and ouabain on lactic production by HeLa cells. The medium was removed from monolayer cultures immediately before the start of the treatment and fresh medium added, together with 1 mM ouabain where indicated. After 4 h at the indicated temperatures the medium from each culture was removed and assayed for lactate as described in **Materials and methods**. Lactic acid produced in control cells (●), lactic acid produced in cells treated with 1 mM ouabain (○). Each point represents the mean of triplicate determinations. Lactic acid excretion into the medium was assayed as described in **Materials and methods**. The bars represent s.d.

HeLa cells over a 4 h period is significantly increased when the cells were incubated at 42°C. Whilst the level of lactic acid accumulation over this period was determined to ensure significant differences, assessment of the time course of lactic acid excretion show the process to be linear up to 4 h, at both 42°C and 37°C. For comparison when the temperature of the incubation was 45°C (under which conditions, protein synthesis ceases and no HSPs are induced), the level of lactic acid excretion was reduced to a level similar to that observed in control cells incubated at 37°C. Also clear from Figure 5 is that inclusion of 1 mM ouabain in the culture medium has an inhibitory effect on lactic acid production. Since 1 mM ouabain completely and specifically inhibits human $Na^+K^+$ATPase by binding to its α-subunit, this observation supports the notion that the $Na^+K^+$ATPase has some role to play in the regulation of glycolysis, at least in HeLa cells. Indeed it could be argued that the addition of ouabain may prevent the increase in lactate production observed at 42°C suggesting that the increase in $Na^+K^+$ATPase activity observed at 42°C (see Table I) may be responsible for the elevated lactate production at 42°C. Since the increase in $Na^+K^+$ATPase observable after treatment at 42°C for 2 h could be inhibited by cycloheximide, the effect of this protein synthesis inhibitor on lactate production over a 2 h period was assessed. However, even at 37°C the addition of cycloheximide, depressed lactate production somewhat (Figure 5). This may result from the

**Figure 5** The effect of cytoheximide on lactic acid production by HeLa cells. After replacement of medium, cells were incubated at 37°C, or 42°C, for 2 h in the presence of 2 μg ml$^{-1}$ cycloheximide (○), 1 mM ouabain (△), 2 μg ml$^{-1}$ cycloheximide plus 1 mM ouabain (□) or without any additions (●). Lactic acid excreted into the medium was assayed as described in **Materials and methods**. The bars represent s.d.

short metabolic half life of one or more glycolytic enzymes. This unfortunately makes interpretation of the other data in Figure 6 difficult.

As an alternative to *continuous* hyperthermia, HeLa cells were exposed to 45°C but for only

**Figure 6** Effect of heat and ouabain on lactate production by human glial and glioma cells. Monolayer cultures of glioma, G-UVW (▲) or glial NOR-P (●) cells were incubated for 4 h at 37°C or 42°C after change of medium in the presence (broken lines) or absence (solid lines) of 1 mM ouabain. The lactic acid excreted into the medium was determined as in **Materials and methods**. Bars represent s.d.

10 min and then returned to 37°C. When lactate production in such cells is measured over a 4 h period at 37°C (after the 2 h required at 37°C for the partial restoration of $Na^+K^+$ATPase) the level is almost exactly that in untreated cells held at 37°C.

*Hyperthermia and lactate production on other human and hamster cells*

Whilst it is clear that $Na^+K^+$ATPase may play a role in regulating glycolysis in HeLa cells and that hyperthermia, at least at 42°C, increases lactate production, an important question is whether a similar situation exists in other cells of human origin, or indeed in cells of other mammalian species. Figure 6 compares the effects of ouabain and hyperthermia on lactate production by a human glioma cell line (G-UVW) and normal glial cells. Incubation at 42°C increases lactate production by the tumour line but this increase is blocked by 1 mM ouabain. On the other hand heat treatment has no effect on lactate production by the normal glial cell line, NOR-P. A similar result was obtained when this experiment was repeated with another pair of glioma and normal glial cell lines, G-CCM and NOR-F.

An increase in lactate production as a result of hyperthermia is also detectable in certain other cultured human cell lines as shown in Table II. Moreover the effect of heat and ouabain are not exclusive to human cells as untransformed baby hamster kidney cells (BHK-21/C13) and polyoma virus transformed (BHK-21/PyY) baby hamster kidney cells show similar responses. Comparison of the lactate produced per unit weight of cellular protein showed that the virus transformed cells have a 35% higher glycolytic rate than their untransformed counterparts, confirming earlier data of Broadfoot *et al.* (1964). However the fact that although the inhibitory effect of ouabain on both the hamster cell lines lends support to the notion of $Na^+K^+$ATPase involvement, this is at variance with the data of Suolinna *et al.* (1974). These authors on the other hand did not measure the effect of ouabain on lactate production in undisturbed, growing monolayers BHK-21 cell cultures in normal medium.

Any far reaching conclusion from the data in Table II would be premature. Although certain

**Table II** Effect of hyperthermia and ouabain on lactate production by other human and hamster cells

| *Cell Type* | *% inhibition of lactate production by 1 mM ouabain* | *% stimulation of lactate production at 42°* |
|---|---|---|
| Chang liver (human) | 52 | 1 |
| FL (human amnion) | 13 | 34 |
| Foetal muscle fibroblasts | 10 | 23 |
| BHK-21/C13 (hamster) | 15 | 11 |
| BHK-21/PyY (polyoma transformed hamster) | 23 | 10 |

After changing the medium, cell cultures were incubated for 2 h at 42° or incubated for 2 h with 1 mM ouabain at 37°C. The lactic acid excreted into the medium was determined in triplicate samples as described in **Materials and methods** and compared with that excreted in 4 h by untreated control cells. The human foetal muscle fibroblasts were a gift from Dr R.L.P. Adams of this Department and were early passage primary cultures. The Chang liver and FL cells were cell lines from Gibco Biocult, Paisley.

neoplastic (glioma), embryonic and foetal cells all show increased lactic acid production at 42°C, a limited selection of normal adult derived cells do not. On the other hand both the transformed and untransformed baby hamster cells show comparable stimulation of lactic acid production at 42°C. It may be important however to consider the origin of the hamster cell line in that it is derived from only 1 or 2 day old animals (Stoker & Macpherson, 1962).

*Hyperthermia and glycolysis in tumour cells*

Although not an obligatory component of the malignant phenotype (Pouyssegur *et al.,* 1980) a prominent feature of certain rapidly growing tumour cells is their capacity to sustain high rates of glycolysis under aerobic conditions. As already discussed the plasma membrane $Na^+K^+$ATPase has been suggested (Racker, 1976) to play some regulatory role in this phenomenon. On the other hand transformation of chick cells by Rous sarcoma virus leads to specific increases in the activities of key enzymes in the glycolytic pathway itself (Singh *et al.,* 1974), and recently certain glycolytic enzymes have been found to become phosphorylated at tyrosine after such viral transformation (Cooper *et al.,* 1983).

Whatever the contributory reasons for the high aerobic glycolytic rate, we find that elevation of the environmental temperature to 42°C under aerobic conditions, leads to a pronounced increase in lactate production in a variety of human cells. Such a phenomenon could conceivably lead to a reduced pH and hence improve the therapeutic effect of hyperthermia against tumour cells. On the other hand a reduced blood supply in the vicinity of a tumour might more realistically result in conditions of oxygen deprivation. Thus any glycolysis might be anaerobic rather than aerobic. Pyruvate produced in glycolysis would have to bypass oxidative phosphorylation mechanisms and be secreted directly as lactate. To assess the effect of inhibition of aerobic respiration on glycolysis the outcome of cyanide addition was determined. From Figure 7 it can be seen that addition of sodium cyanide to the medium had really little effect on lactate production in HeLa cells at 37°C.

This is surprising as reduction in ATP production by inhibition of respiration might be expected to be compensated by increased glycolysis. Tight control of ATP levels in certain mammalian cells is believed to be mediated by the activity of adenylate kinase which serves to amplify the effects of small changes in ATP by converting them to large proportional changes in AMP (Newsholme & Start, 1979). Such changes in AMP would then overcome the previously inhibitory effect of ATP on phospho-

**Figure 7** Effect of heat and the presence of sodium cyanide on lactate production by HeLa cells. After replacement of the medium, cultures of HeLa cells were incubated for 4 h at various temperatures in the presence (△) or absence (●) of 5 mM sodium cyanide. The lactate excreted into the medium was determined as described in **Materials and methods**. Bars represent s.d.

fructokinase. However such a mechanism may be inefficient in our HeLa cells (see Lunec & Cresswell, 1983), or mitochondria "poisoned" with such a respiratory inhibitor might release accumulated $Ca^{2+}$ ions (Drahota *et al.,* 1965; Carafoli, 1974) which could depress glycolysis through effects on pyruvate kinase (Meli & Bygrave, 1972). Alternatively cyanide treatment may bring into play a cryptic lactate dehydrogenase. Recently it has been shown that a considerable number of human carcinomas are associated with massively elevated levels of a cryptic lactate dehydrogenase (Anderson & Kovacik, 1981). The activity of this unusual lactate dehydrogenase ($LDH_K$), is also readily detectable in HeLa cells, but it is inhibited by physiological concentrations of oxygen (Anderson *et al.,* 1981). *In vitro* the enzyme can only be assayed either under nitrogen or in the presence of 5 mM sodium cyanide, which activates it (Anderson *et al.,* 1981). The enzyme may contain multiple haem groups (Anderson *et al.,* 1981) and thus have some similarities with yeast cytochrome lactate dehydrogenase, cytochrome $b_5$ (see Hatefi & Stigall, 1976). The function of this particular yeast enzyme is unclear but it may be involved in shifting from aerobic to anaerobic metabolism.

Further inspection of Figure 7 shows that if the temperature of the HeLa cell cultures incubated with cyanide is raised to 42°C the output of lactic acid declines markedly, unlike the situation in cells incubated without cyanide. This observation is compatible with the notion that $LDH_K$ may become an important regulatory factor in the cyanide treated cells, but that it is adversely affected by heat. The *in vitro* data of Anderson *et al.* (1981) appear to show that $LDH_K$ has a half-life at 42°C in the presence of cyanide of only ~4 min. Thus in the presence of cyanide it may be that heat has a specific adverse effect on HeLa cell lactic acid production possibly through effects on $LDH_K$. Whilst high levels of $LDH_K$ are associated with many human cancers (Anderson & Kovacik, 1981) $LDH_K$ can be induced in rat fibroblasts subject to anoxia (Anderson *et al.*, 1979), and thus expression of $LDH_K$ in human cancer may reflect such stress. Alternatively constitutive expression of $LDH_K$ might confer on tumour cells the ability to proliferate under low oxygen tension, by maintaining a proper redox potential without oxygen (Anderson & Kovacik, 1981). This apparently convenient situation may however be impaired at elevated temperatures due to the thermal instability of the $LDH_K$ and lead to depressed tumour survival. It should be stressed however that whilst the adverse effects of heat on the cyanide-treated cells may be due to effects on a vulnerable $LDH_K$, cyanide treatment is not equivalent to hypoxia or anoxia.

Since the above proposal does not take into account the continued presence of normal LDH isoenzymes alternative explanations should be considered. For instance as already suggested treatment with certain respiratory inhibitors might impair the maintenance of mitochondrially stored $Ca^{2+}$. Such an impairment may simply increase with hyperthermia leading to even greater depression of glycolysis. This alternative notion is supported by the observation that almost identical data can be obtained from HeLa cells treated with hyperthermia but in the presence of a 1 $\mu$M concentration of the respiratory uncoupler, FCCP (carbonyl cyanide p-trifluoromethoxyphenylhydrazone).

### References

ANDERSON, G.R. & KOVACIK, W.P. (1981). $LDH_K$, an unusual oxygen sensitive lactate dehydrogenase expressed in human cancer. *Proc. Natl Acad. Sci.*, **78**, 3209.

ANDERSON, G.R., KOVACIK, W.P. & MAROTTI, K.R. (1981). $LDH_K$, an uniquely regulated cryptic lactate dehydrogenase associated with transformation with Kirsten Sarcoma Virus, *J. Biol. Chem.*, **256**, 10583.

ANDERSON, G.R., MAROTTI, K.R. & WHITAKER-DAWLING, P.A. (1979). A candidate rat-specific gene product of the Kirsten Murine Sarcoma virus. *Virology*, **99**, 31.

ANDERSON, K.C., MINTON, K.W. & LI, G.C. (1981). Temperature induced homeoviscous adaptation of Chinese hamster ovary cells. *Biochem. Biophys. Acta*, **641**, 334.

BELT, J.A., THOMAS, J.A., BUCHSBAUM, R.N. & RACKER, E. (1979). Inhibition of lactate transport and glycolysis in Ehrlich ascistes tumour cells by bioflavenoids. *Biochemistry*, **18**, 3506.

BONANOU-TZEDAKI, S.A., SOHL, M.K. & ARNSTEIN, H.R.V. (1981). Regulation of protein synthesis in reticulocytes: characterisation of the inhibitor generated in the post-ribosomal supernatant by heating at 44°C. *Eur. J. Biochem.*, **14**, 69.

BROADFOOT, M., WALKER, P., PAUL, J., MACPHERSON, I. & STOKER, M. (1964). Glycolysis and respiration of transformed BHK-21 cells. *Nature*, **204**, 79.

BROTHERUS, J.R., JOST, P.C., GRIFFITH, O.H., KEANA, J.F. & HOKIN, L.E. (1980). Charge selectivity at the lipid-protein interface of membranous $Na^+K^+$ATPase, *Proc. Natl Acad. Sci.*, **7**, 272.

BURDON, R.H. & CUTMORE, C.M.M. (1982). Human heat shock gene expression and the modulation of plasma membrane $Na^+K^+$Pase activity. *FEBS Letts.*, **140**, 45.

BURDON, R.H., SLATER, A., MCMAHON, M. & CATO, A.C.B. (1982). Hyperthermia and the heat shock proteins of HeLa cells. *Br. J. Cancer*, **45**, 953.

CARAFOLI, E. (1974). Mitochondrial uptake of calcium ions and the regulation of cell function. *Biochem. Soc. Symp.*, **39**, 89.

CATO, A.C.B., ADAMS, R.L.P. & BURDON, R.H. (1978). Genome modification in two lines of baby hamster kidney fibroblasts (BHK-21). *Biochim. Biophys. Acta*, **521**, 397.

COOPER, J.A., REISS, N.A., SCHWARTŻ, R.J. & HUNTER, T. (1983). Three glycolytic enzymes are phosphorylated at tyrosine in cell transformed by Rous sarcoma virus. *Nature*, **302**, 218.

CRESS, A.E. & GERNER, E.W. (1980). Cholesterol levels inversely reflect the thermal sensitivity of mammalian cells in culture. *Nature*, **283**, 677.

DRAHOTA, Z., CARAFOLI, E., ROSSI, C.S., GAMBLE, R.L. & LEHRINGER, A.L. (1965). The steady state maintenance of accumulated $Ca^{2+}$ in rat liver mitochondria. *J. Biol. Chem.*, **240**, 2712.

EDIDIN, M. (1974). Rotational and translational diffusion in membranes. *Annu. Rev. Biophys. Bioeng.*, **3**, 179.

FIELD, S.B. & ANDERSON, R.L. (1982). Thermotolerance: a review of observations and possible mechanisms. *Natl Cancer Inst. Monogr.*, **61**, 193.

FIELD, S.B. & BLEEHAN, N.M. (1979). Hyperthermia in the treatment of cancer. *Cancer Treat. Rev.*, **6**, 63.

GERWECK, L.E. (1977). Modification of cell lethality at elevated temperature. The pH effect. *Radiat. Res.*, **70**, 224.

GERWECK, L.E., JENNINGS, M. & RICHARDS, B. (1980). Influence of pH on the response of cells to single and split doses of hyperthermia. *Cancer Res.*, **40**, 4019.

GULLINO, P.M., GRANTHAM, F.H., SMITH, S.H. & HAFFERTY, A.C. (1965). Modifications of the acid-base status of the internal milaeu of tumours. *J. Natl Cancer Inst.*, **34**, 857.

HATEFI, Y. & STIGALL, D.L. (1976). Metal-containing flavoprotein dehydrogenases, in *The Enzymes* (Ed. Boyer), New York: Academic Press, vol. 13 p. 175.

HAZEL, J.R. & PROSSER, C.L. (1974). Molecular mechanisms of temperature compensation in poikilotherms. *Physiol. Rev.*, **54**, 620.

HENLE, K.J., KARAMUZ, J.E. & LEEPER, D.B. (1978). Induction of thermotolerance in Chinese Hamster Ovary Cells by high (45°) or low (40°) hyperthermia. *Cancer Res.*, **38**, 570.

HICKEY, E.D. & WEBER, L.A. (1982). Modulation of heat shock polypeptide synthesis in HeLa cells during hyperthermia and recovery. *Biochemistry*, **21**, 1513.

HUGHES, E.N. & AUGUST, J.T. (1982). Coprecipitation of heat shock proteins with a cell surface glycoprotein. *Proc. Natl Acad. Sci.*, **79**, 2305.

JOHNSON, S., STOKKE, T. & PRYDZ, H. (1975). HeLa cell plasma membranes 1.5′-nucleotidase and ouabain-sensitive ATPase as markers for plasma membranes. *J. Cell Biol.*, **63**, 357.

LI, G.C. & WERB, Z. (1982). Correlation between synthesis of heat shock proteins and development of thermotolerance in Chinese hamster fibroblasts. *Proc. Natl Acad. Sci.*, **79**, 3218.

LUNEC, J. & CRESSWELL, S.R. (1983). Heat induced thermotolerance expressed in the energy metabolism of mammalian cells. *Radiat Res.*, **93**, 588.

McDONOUGH, J.P. & MAHLER, H.P. (1982). Covalent phosphorylation of the $Mg^{2+}$-dependent ATPase of yeast plasma membranes. *J. Biol. Chem.*, **257**, 14579.

MELI, J. & BYGRAVE, F.L. (1972). The role of mitochondria in modifying calcium sensitive cytoplasma metabolic activities. Modification of pyruvate kinase activity. *Biochem. J.*, **128**, 415.

MYER, K.A., KAMMERLING, E.M., AMTMAN, L., KILLER, M. & HOFFMAN, S.J. (1948). pH studies of malignant tissues in human beings. *Cancer Res.*, **8**, 513.

NAESLUND, J. & SWENSON, K.E. (1953). Investigation on the pH of malignant tumours of mice and humans after the administration of glucose. *Acta. Obstet. Gynecol.* (*Scand.*), **32**, 359.

NEWSHOLME, E.A. & START, C. (1979). *Regulation in Metabolism*, Wiley, New York p. 111.

OVERGAARD, J. & BICHEL, P. (1977). The influence of hypoxia and acidity on the hyperthermic response in malignant cells *in vitro*. *Radiology*, **123**, 511.

POUYSSEGUR, J., FRANCHI, A., SALOMON, J.-C. & SILVESTRE, P. (1980). Isolation of Chinese hamster fibroblast mutant defective in hexose transport and aerobic glycolysis: its use to dissect the malignant phenotype. *Proc. Natl Acad. Sci.*, **77**, 2698.

RACKER, E. (1976). *A New Look at Mechanisms in Bioenergetics*. New York: Academic Press.

RACKER, E., JOHNSON, J.H. & BLACKWELL, M.T. (1983). The role of ATPase in glycolysis of Ehrlich ascites tumour cells. *J. Biol. Chem.*, **257**, 3702.

RATTRAY, J.B., SCHIBECI, A. & KIDBY, D.K. (1975). Lipids of yeasts. *Bacteriol Rev.*, **39**, 197.

SCHLESINGER, M.J., ALPERTI, G. & KELLEY, P.M. (1982). The response of cells to heat shock. *Trends Biochem. Sci.*, **7**, 222.

SCHOLNICK, P.I., LANG, D. & RACKER, R. (1973). Regulations mechanisms in Carbohydrate metabolism IX Stimulation of aerobic glycolysis by energy linked in transport and inhibition by dextran sulphate. *J. Biol. Chem.*, **248**, 5175.

SINGH, M., SINGH, V.N., AUGUST, J.T. & HORECKER, B.L. (1974). Alterations in glucose metabolism in Chick Embryo Cells transformed by Rous Sarcoma Virus. Transformation specific changes in the activities of key enzymes of the glycolytic and hexose monophosphate shunt pathways. *Arch. Biochem. Biophys.*, **165**, 240.

STOKER, M. & MACPHERSON, I.A. (1962). Studies on transformation of hamster cells by polyoma virus *in vitro*. *Virology*, **14**, 359.

STROBEL, G.A. (1979). The relationship between membrane ATPase activity in sugar cane and heat induced resistance to helminthosporide. *Biochim. Biophys. Acta*, **554**, 460.

SUOLINNA, E.-M., LANG, D.R. & RACKER, E. (1974). Quercitin, an artificial regulator of the high aerobic glycolysis of tumour cells. *J. Natl Cancer Inst.*, **53**, 1515.

TANAKA, R. & TERUYA, A. (1973). Lipid dependence of activity-temperature relationship of $Na^+K^+$ activated ATPase. *Biochim. Biophys. Acta*, **323**, 584.

WANG, C., GOMER, R.H. & LAZARIDES, E. (1981). Heat shock proteins are methylated in avian and mammalian cells. *Proc. Natl Acad. Sci.*, **78**, 3531.

WANG, C., ASAI, D.J. & LAZARIDES, E. (1980). The 68,000-dalton neurofilament-associated polypeptide is a component of nonneuronal cells and of skeletal myofibrils. *Proc. Natl Acad. Sci.*, **77**, 1541.

WARBURG, O. (1926). *Uber den Stoffwechsel der Tumoren*, Berlin: Springer Verlag, p. 00.

YEH, L.-A., LING, L., ENGLISH, L. & CANTLEY, L. (1983). Phosphorylation of the $Na^+K^+$ATPase by a plasma membrane-bound protein kinase in Friend Erythroleukaemia cells. *J. Biol. Chem.*, **258**, 6567.